# Transmission-blocking activity of antibodies to *Plasmodium falciparum* GLURP.10C chimeric protein formulated in different adjuvants

**DOI:** 10.1186/s12936-015-0972-0

**Published:** 2015-11-09

**Authors:** Will Roeffen, Michael Theisen, Marga van de Vegte-Bolmer, GeertJan van Gemert, Theo Arens, Gorm Andersen, Michael Christiansen, Laxman Sevargave, Shrawan Kumar Singh, Swarnendu Kaviraj, Robert Sauerwein

**Affiliations:** Department of Medical Microbiology, Radboud University Medical Center, Nijmegen, The Netherlands; Department for Congenital Disorders, Statens Serum Institut, Copenhagen, Denmark; Department of International Health, Immunology, and Microbiology, Centre for Medical Parasitology, University of Copenhagen, Copenhagen, Denmark; Department of Infectious Diseases, Copenhagen University Hospital, Rigshospitalet, Copenhagen Denmark; Gennova Biopharmaceuticals Limited, Pune, India

**Keywords:** *Plasmodium falciparum* malaria, R0.10C immunogenicity, Alhydrogel, Glucopyranosyl Lipid Adjuvant (GLA), *Pf*s48/45 vaccine candidate, Standard Membrane Feeding Assay (SMFA)

## Abstract

**Background:**

*Plasmodium falciparum* is transmitted from person to person by *Anopheles* mosquitoes after completing its sexual reproductive cycle within the infected mosquito. An efficacious vaccine holds the potential to interrupt development of the parasite in the mosquito leading to control and possibly eradication of malaria. A multi-component, R0.10C, was developed comprising *P. falciparum* glutamate-rich protein (R0) fused in frame to a correctly folded fragment of *Pf*s48/45 (10C). Here, a series of novel adjuvants were screened for their ability to elicit transmission-blocking (TB) antibodies.

**Methods:**

The recombinant fusion protein R0.10C was produced in *Lactococcus lactis* and purified by affinity-chromatography on a monoclonal antibody (mAb 85RF45.1) against a major epitope for TB antibodies (epitope 1) harboured on R0.10C. Immune-purified R0.10C was mixed with a series of adjuvants and tested in mice and rats.

**Results:**

In general, all R0.10C formulations elicited high levels of antibodies recognizing native *Pf*s48/45 in macrogametes/zygotes. TB activity of anti-R0.10C antisera was assessed in the standard membrane-feeding assay (SMFA). Potency of different adjuvant/R0.10C combinations was tested in mice and rats using aluminium hydroxide (Alum), Alum with micellar and emulsion formulations of a synthetic TLR4 agonist, Glucopyranosyl Lipid Adjuvant (GLA), stable emulsion (SE)/GLA, AbISCO-100 and Freund’s adjuvant (as reference). All formulations produced high antibody titres recognizing the native Pfs48/45 protein in macrogametes/zygotes. Interestingly, the GLA-Alum combination adjuvant was the most potent inducer of TB antibodies based on serum collected after two immunizations. In agreement with previous observations, biological activity in the SMFA correlated well with the level of anti-*Pfs*48/45 antibodies.

**Conclusion:**

The combined data provide a strong basis for entering the next phase of clinical grade R0.10C production and testing.

## Background

Malaria is primarily confined to the poorest tropical areas of the world and constitutes one of the world’s greatest public health problems [1]. Efforts to eliminate malaria transmission through use of insecticides and/or bed nets have resulted in only limited success [2]. Vaccines hold the potential for effective control and elimination of the disease burden. However, despite decades of research there is still no clinically applicable vaccine available. With the renewed focus on malaria eradication, transmission blocking malaria vaccines (TBMV) which induce antibodies that target the sexual stages of the parasite have become a focus of malaria vaccine research. One of the leading candidates for a TBMV is *Pf*s48/45, a protein expressed in gametocytes and on the surface of gametes and zygotes [3–6]. *Pf*s48/45 is essential for male gamete fertility in *Plasmodium* [7] and anti-*Pf*s48/45 antibodies have been found to prevent zygote development in the standard membrane feeding assay (SMFA) [8]. The exact mechanism through which these antibodies interfere with fertility is unknown but steric hindrance is likely a possibility. Irrespective of the mechanism, it has been shown in ex vivo assays that there is a strong correlation between levels of *Pf*s48/45-specific antibodies in sera from malaria-endemic areas and TB activity in the SMFA [9–11].

*Pf*s48/45 is a relatively cysteine-rich protein with multiple disulfide bonds resulting in antibody epitopes that are dependent on tertiary structure rather than linear amino acid sequence. A panel of mAbs have been generated and characterized with respect to their TB-activity in SMFA [12, 13]. Of these, mAb 85RF45.1 exhibits the strongest TB activity whereas mAbs 85RF45.2 and 85RF45.3 do not exhibit TB activity on their own but may act synergistically with 85RF45.1 [12]. All three mAbs recognize conformational epitopes, of which epitope I, defined by mAb 85RF45.1, is located in the C-terminal portion of *Pf*s48/45 (10C) [14]. *Pf*s48/45 and fragments thereof has been produced on recombinant form in various expression systems [5, 6, 15–18]; however, none of these products has reached clinical development phase. Recently, correctly folded fragments were produced of *Pf*s48/45 (10C) containing all three epitopes and *Pfs*48/45 (6C) containing only TB-epitope 1 fused in frame with the N-terminal part of the glutamate rich protein (GLURP) in the *Lactococcus lactis* expression system [19, 20]. This system has previously proved efficient for the production of GMZ2, an asexual blood-stage vaccine candidate [21, 22], and GMZ2 adjuvanted in Al(OH)_3_ has shown good safety and tolerability in phase 1 clinical trials [21, 23–25].

With the aim of developing a TBMV, the effects of different adjuvant formulations were investigated in mice and rats on: (1) the production of antigen-specific IgG against recombinant and native proteins; and, (2) the biological activity of antibodies elicited by vaccination. The adjuvant vehicles used were either aluminium hydroxide (Alum), an oil-in-water stable emulsion (SE) or AbISCO^®^-100, a saponin based adjuvant. Two of these formulations (Alum and SE) were supplemented with the Toll-like receptor synthetic TLR 4 agonist glucopyranosyl lipid A adjuvant (GLA) [26]. GLA was used in the present study because it has been shown to enhance IgG responses against the GLURP.R0 component of GMZ2 in mice [27], and GLA is safe for use in humans and non-human primates [28].

## Methods

### Construction, fermentation and purification of correctly folded R0.10C

Construction of the recombinant R0.10C hybrid molecule, a fusion protein containing the regions GLURP_27–500_ and *Pf*s48/45_159–__428_ and a stretch of six histidines in the C-terminus, has been described in detail elsewhere [19]. The theoretical molecular weight of the protein is 89.8 kDa of which 30.7 kDa originates for the 10C fragment.

All fermentations at the 40L scale were done at the cGMP-facility of Gennova, India, using the standard proposed medium composition as described earlier [19]. Briefly, a fermentor containing reconstituted medium with the final pH 7.0, adjusted with sterile 2 N NaOH, was inoculated with overnight culture of inoculum. The fermenter was programmed to maintain pH 6.5, temperature 30 °C at the low agitation rate (100 rpm) without aeration, maintaining anaerobic conditions. At the stationary phase when no more alkali (2 N NaOH) uptake was observed to maintain pH 6.5, the culture was harvested from the fermenter. Following clarification of the fermentation broth by centrifugation, the culture supernatant was concentrated five to six times using tangential flow filtration (TFF) and diafiltered using 2.5 sq m membrane with pore size cut-off of 10 kDa with buffer-1 (50 mM phosphate buffer, pH 7.0, 6 diafiltration volumes (DV) followed by buffer-2 (50 mM phosphate buffer, pH 7.0 and 0.25 M NaCl, 6 DV). The diafiltrate, now defined as ‘affinity-input’ containing 10 mM imidazole and 5 mM β-ME, was loaded on the Ni2+-Sepharose column pre-equilibrated with 4CV of equilibration buffer (50 mM phosphate buffer, pH 7.0, 0.25 M NaCl, 20 mM Imidazole). After loading affinity-input, the column was washed with 4 column volumes (CV) of equilibration buffer, followed by washing with redox-buffer of glutathione exists in both 4 mM reduced (GSH) and 0.4 mM oxidized (GSSG) states for 60 min. Following redox-wash, the recombinant R0.10C was eluted isocratically with the elution buffer containing 50 mM phosphate buffer, 0.25 M NaCl, 200 mM Imidazole, pH 7.0. The peak fraction was finally purified on anti-*Pf*s48/45 mAb 45.1 (epitope 1) coupled to agarose using the AminoLink^®^ Plus Immobilization Kit (Pierce: 44894) according to the instructions of the manufacturers. The eluate contained immune purified R0.10C (correctly folded) whereas the run-through contained <1 % of correctly folded R0.10C. Eluted samples were concentrated to at least 1 mg/ml against 1× phosphate buffered saline (PBS) on a Vivaspin20, 30-kDa molecular mass cut-off ultra-filtration unit (Vivascience, Germany). Protein concentrations were measured on a NanoDrop 2000 (Thermo Scientific, USA) using a molar extinction coefficient of 0.3 and Bradford’s method.

### Rat and mouse immunogenicity studies

Experimental animals, including female Wistar Hanover rats and female CB6F1 (BALB/c × C57BL/6 F1 hybrid) mice, were selected for the study. The animals were purchased from Charles River and kept in the Laboratory Animal Facility Centre of the Radboud University Medical Centre (RUMC). All procedures regarding animal immunization complied with European and national regulations.

Immunizations were performed by the subcutaneous route three times, 2 weeks apart with 20 µg of R0.10C adjuvanted with different formulations in a total volume of 100 µl for mice and 200 µl for rats.

Rats receiving adjuvant formulations aluminium hydroxide [(Alhydrogel, Brenntag (Alum: SSI (expiry date June 2012)], Alum/GLA, SE/GLA and AbISCO-100 and for mice complete Freund’s adjuvant (CFA)/incomplete Freund’s adjuvant (IFA) (Sigma-Aldrich), Alum/GLA, SE/GLA and AbISCO-100 (ISCONOVA). The Freund’s group was used for reasons of comparison with the former MBP-PF10C immunizations previously described [6]. For the Alum-based formulation, groups of five animals were immunized with R0.10C adsorbed to Alum with or without a supplement of 5 µg of an aqueous formulation of GLA (IDRI:AQ014). For the SE-based immunization, groups of five animals were immunized with R0.10C and SE (IDRI:EM030) supplemented with 5 µg of GLA in SE (IDRI:EM031). The components of the Alum and SE-based formulations were mixed gently for 30 min and allowed to rest on ice for 1 h before injection. Sera were collected from peripheral blood samples on days 0, 14, 28, and 42 separated by centrifugation of blood at 1500×*g* for 10 min and stored at −20 °C until further analysis.

### ELISA

Antibody responses in immunized animals were measured by ELISA. All experimental steps, except for coating, were performed at room temperature (RT). Briefly, ELISA MaxiSorp microtitre plates (Sterilin^®^ ELISA plates, The Netherlands) were coated at 4 °C overnight with 50 μl of the R0.10C (2 µg/ml), GLURP.RO (1.4 µg/ml) or MBP.10C (2 µg/ml) in PBS pH 7.4. Wells were then blocked with 5 % skimmed milk powder in PBS for 1 h followed by three washings with PBS containing 0.05 % Tween-20 (PBST). Plates were incubated with 50 μL two-fold serial dilutions of sera starting with 1:100 in PBST for 4 h at RT. After washing, the plates were incubated with 50 μL of 1:3000 diluted rabbit-anti rat IgG-HRP (H + L) (DAKO, 1:3,000 diluted in PBST) or rabbit anti-mouse IgG-HRP (DAKO, 1:4,000 diluted in PBST) for 2 h. Wells were washed with PBS and subsequently incubated with tetramethyl benzidine (TMB) substrate solution for 20 min. The colour reaction was stopped with 0.2 N H_2_SO_4_, and the optical density (OD) was read at 450 nm in an Anthos 2001 Microplate Reader (Labtec BV). Midpoint (EC50) values were calculated using GraphPad Prism, (GraphPad Software, USA). MBP.10C is used for comparison of results with the data as described by Outchkourov et al. [6]. The discrepancy between the antibody titer measured in the R0.10C and MBP.10C ELISAs is due to antibodies against the R0 region of R0.10C.

### Standard membrane feeding assay (SMFA)

Antisera obtained from mice and rats immunized with R0.10C were assessed for TB activity by SMFA as previously described [29, 30]. Briefly, 30 μl of rat or mouse serum was mixed with 90 μl of naïve human serum and 150 μl of in vitro gametocyte cultures of the *P. falciparum* (NF54) and for comparison (NF135.C10 Cambodian line [31]) laboratory lines. The mixture was fed to *Anopheles stephensi* mosquitoes through a membrane feeding apparatus. Pre-immune sera served as the controls. Fully engorged mosquitoes were separated and held at 26 °C. Seven days later, midguts of 20 mosquitoes were examined for oocysts. The observed TB activity of serum (TRA) was determined as the percentage reduction in the arithmetic mean oocyst number in test samples compared to that in controls. The experiment was considered valid when at least 85 % of the mosquitoes feeding on control sera were infected. Data were analysed by a non-parametric test by comparing the medians of two groups using the Mann–Whitney test, or by comparing three or more groups using the Kruskal–Wallis test, followed by post-tests. If significance was indicated, Dunn’s analysis was used for comparison with the control group.

## Results

### Purification of properly folded R0.10C

**Fig. 1:**
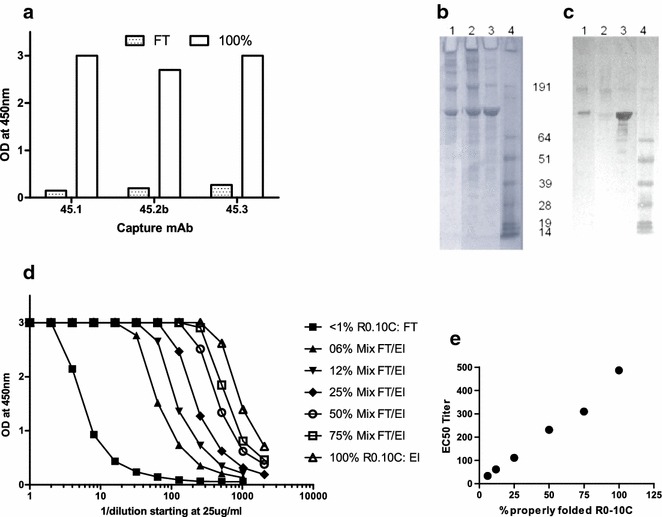
R0-10C purified by mAb45.1-affinity chromatography. Purification and characterization of correctly folded monomeric R0.10C. **a** 2-site ELISA with the flow trough (FT) and immune purified antigen (500 ng/well) using mAb 85RF45.1, 85RF45.2b and 85RF45.3 as capture and anti-HIS-HRPO for detection. **b** Coomassie blue-stained 4–12 % Bis–Tris gel of R0.10C purification steps using 5 µg antigen/lane, **c** Western blot analysis using rat mAb 85RF45.1-HRP for detection. Lane 1, input antigen after Ni^2++^ column purification; Lane 2, Flow trough (FT: misfolded R0.10C) from the mAb Affinity column: lane 3, eluate (immune purified R0.10C (properly folded)) from the mAb affinity column; Lane 4, Markers, the sizes (kDa) of the molecular mass markers are indicated. **d** 2-site ELISA with the FT, mAb-Affinity eluted antigen (El) and mixed samples using mAb 85RF45.1 as capture and mAb 85RF45.2b-HRPO for detection. **e** correlation between EC50 titres and percentage of properly folded antigen (R = 0.9944: y = 4.59x + 0.505)

Recently, a correctly folded fragment was produced of *Pf*s48/45 (10C) as a chimera (R0.10C) fused in frame with a section of the *P. falciparum* glutamate rich protein (GLURP.R0) in the *Lactococcus lactis* expression system [19]. R0.10C was immune-purified on mAb 85RF45.1 bound to agarose beads. The resulting immune-purified R0.10C reacted strongly with mAbs against the conformational epitopes 1, 2b and 3 as demonstrated by sandwich ELISA (Fig. 1a) suggesting that this preparation contains a homogeneous solution of correctly folded R0.10C. In contrast, the flow-through (FT) did not show an appreciable binding to any of the mAbs (Fig. 1a–c) and only affinity-purified R0.10C showed strong reactivity with mAb 85RF45.1 by Western blotting (Fig. 1c). To establish a standard reference curve for estimating the percentage of correctly folded protein species in a solution, immune-purified R0.10C (termed 100 % correctly folded) was mixed with the FT containing only misfolded antigen to obtain mixtures of 100, 75, 50, 25, 12, 6.5, and <1 %, correctly folded R0.10C, respectively. When each mixture was tested in the sandwich ELISA, there was a strong correlation between the EC50 values and percentage of properly folded antigen (Fig. 1d, r = 0.99). Using this reference, the Ni-IMAC purification step yields a product which contains approximately 5 % correctly folded conformers (Fig. 1d).

### Immunogenicity of properly folded R0.10C

**Fig. 2:**
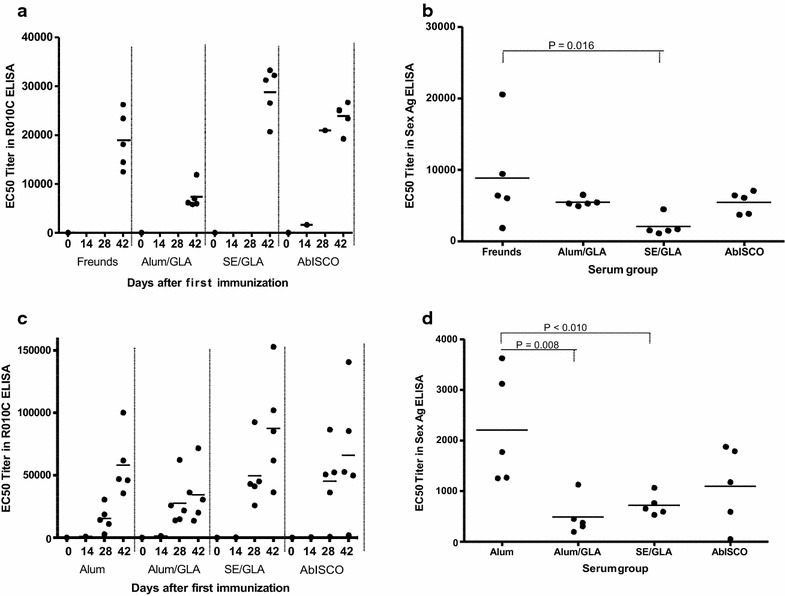
Immunogenicity of R0.10C. ELISA results of various bleed sera from mice (**a** **b** *upper graphs*) and rats (**c**, **d** *lower graphs*) immunized with R0.10C and different adjuvants. 0, 14, 28, and 42 indicate pre-immune sera, day 14, day 28 and day 42 of the time course of the immunization for mouse (**a**) and rat (**c**). Mouse sera (**a**) from day 14 and 28 were not available. The right figures are ELISA results of final bleed (day 42) sera from mice (**b**) and rats (**d**) immunized with immune purified R0.10C formulated with different adjuvants using 10C as sexual stage antigen (Sex Ag)

In order to study the immunogenicity of R0.10C, different adjuvants formulations were investigated in both mice and rats. Inbred CB6F1 (BALB/c x C57BL/6 F1 hybrid) mice were immunized three times at two-week intervals with 20 µg of immune-purified R0.10C administered in AbISCO, Alum supplemented with GLA, or in an oil-in-water SE supplemented with GLA. For comparison, a control group received R0.10C in Freund’s adjuvant. Sexual blood-stage, specific antibody responses were measured by ELISA. Two weeks after the last injection (day 42), all mice had relatively high antibody titres against the immunogen (Fig. 2a). When assessing the IgG responses against the *Pf*s48/45 components, it was evident that R0.10C in SE supplemented with GLA gave the lowest and significantly different (p = 0.016) anti-*Pf*s48/45 IgG response compared to the control group (Fig. 2b). In contrast, R0.10C in Alum supplemented with GLA gave a relatively high anti-*Pf*s48/45 IgG response indistinguishable (p = 0.3) from the response obtained with R0.10C in Freund’s adjuvant (Fig. 2b). The finding that an Alum-based formulation is as efficient as an oil-in-water emulsion was unexpected and warrants further investigation. A second experiment, including an additional group of R0.10C/Alum, was therefore performed in rats. Rats were chosen partly to confirm the initial finding in a second species and partly because rats can donate more blood, allowing analysis of individual samples in functional bioassays. Animals were immunized three times with 20 µg immune purified R0.10C adjuvanted in Alum, Alum/GLA, SE/GLA or AblSCO (Fig. 2c). Compared with the mouse experiment, R0.10C adjuvanted in Alum/GLA gave the lowest anti-10C IgG levels and is significantly different from the Alum group (p = 0.008). Furthermore, R0.10C adjuvanted in Alum gave the highest anti-*Pf*s48/45 IgG response and is also significantly different from the SE/GLA group (p < 0.01) but not from the AbISCO group (p > 0.2) (Fig. 2d).

Antibodies against GLURP.R0 showed that the lowest antibody titers (EC50 titer = 1984 with standard deviation (SD) of 889) was detected for the alum group but is only significantly different (p = 0.01) from the SE/GLA and AbISCO group. There was no differences in antibody titers between the Alum/GLA, SE/GLA and AbISCO group; EC50 titers 4776 (SD = 2419), 6371 (SD = 4093) and 7462 (SD = 4098), respectively.

### Inhibition of sexual *Plasmodium falciparum* fertilization by R0.10C antisera

TB activity of anti-R0.10C antisera was assessed in the SMFA. The pre-bleed (day 0) of mice and rats did not show any TB activity and was used to calculate the per cent inhibition of oocyst counts. Two injections of R0.10C formulated in Alum/GLA or AbISCO elicit IgG levels which efficiently inhibited (>96 %) oocyst development, whereas three injections of R0.10C in SE/GLA and Freund’s were required to reach the same level of inhibition (Table 1). The same trend was also observed in rats (Table 1).

**Table 1:** Infectivity of *P. falciparum* Gametocytes to *A. stephensi* Mosquitoes in the presence of different pooled bleed sera from mice and rats immunized with R0.10C formulated with different adjuvants in the Standard Membrane-Feeding Assay (SMFA)

Day	Mice								Rats							
	Freunds		Alum/GLA		SE/GLA		AbISCO		Alum		Alum/GLA		SE/GLA		AbISCO	
	Inf	TRA, %	Inf	TRA, %	Inf	TRA, %	Inf	TRA, %	Inf	TRA, %	Inf	TRA, %	Inf	TRA, %	Inf	TRA, %
0	18		18		19		18		16		17		19		16	
14	17	0	11	35	11	26	18	0	20	11	17	2	20	0	17	0
28	7	76	1	97	10	59	4	96	5	94	3	97	10	85	6	91
42	0	100	2	95	1	99	1	99	1	98	1	99	1	99	1	99

Pooled Sera (1:9 diluted) from the different bleeds (Day 0, 14, 28 and 42) were tested in the SMFA. Seven days after membrane feed, the number of infected mosquitoes and oocyst density were determined. Inf: total infected mosquitoes from the 20 dissected mosquitoes; TRA, %: percentage of Transmission Reducing Activity using the pre-bleed (Day 0) sample for calculation

When testing individual day 42 sera from rats, the TB activity of the four adjuvant formulations resulted in >99 % inhibition of oocyst development except for one rat in the Alum/GLA (TRA 25 %) and one in the AbISCO group (TRA 29 %) (Table 2). These two non-blocking antisera also gave the lowest anti-*Pfs*48/45 IgG titres (196 and 51 respectively).

**Table 2:** Infectivity of *P. falciparum* Gametocytes to *A. stephensi* Mosquitoes in the presence of day 42 serum from rat immunized with R0.10C formulated with different adjuvant formulation in the Standard Membrane Feeding Assay (SMFA

Group	Rat	Inf	Mean (SD)	%	*P* value
Alum	1	1	0.05 (0.22)	99	<0.001
	2	0	0.0 (0.0)	100	<0.001
	3	0	0.0 (0.0)	100	<0.001
	4	1	0.05 (0.22)	99	<0.001
	5	0	0.0 (0.0)	100	<0.001
Alum/GLA	6	0	0.0 (0.0)	100	<0.001
	7	1	0.05 (0.22)	99	<0.001
	8	18	7.85 (4.64)	25	NS
	9	1	0.05 (0.22)	99	<0.001
	10	1	0.05 (0.22)	99	<0.001
Pre sera		37/40	10.48 (6.12)		
SE/GLA	11	2	0.10 (0.31)	99	<0.001
	12	2	0.10 (0.31)	99	<0.001
	13	1	0.05 (0.22)	99	<0.001
	14	1	0.05 (0.22)	99	<0.001
	15	0	0.0 (0.0)	100	<0.001
AbISCO	16	0	0.0 (0.0)	100	<0.001
	17	16	7.45 (6.39)	29	NS
	18	0	0.0 (0.0)	100	<0.001
	19	1	0.05 (0.22)	99	<0.001
	20	2	0.10 (0.31)	99	<0.001

Sera (1:9 diluted) were tested in the SMFA. Seven days after membrane feed, the number of infected mosquitoes and oocyst density were determined. Sera: Rat serum from R0.10C-immunized group; Control: feeder with pre-bleed serum (day 0) of the group; no. Inf: total infected from 20 dissected mosquitoes; TRA, %: percentage of Transmission Reducing Activity; P-value: sample of the control (day 0) was used for calculation

*NS* not significant; *SD* standard deviation

### R0.10C antisera inhibit *Plasmodium falciparum* growth in a strain independent manner

Antigenic variation may limit the breadth of *P. falciparum*-inhibiting antibodies. To gauge the breadth of *P. falciparum* inhibition antisera from all animals immunized with R0.10C/Alum was tested for TB activity of genetically diverse African and Cambodian *P. falciparum* laboratory lines NF54 and NF135.10C, respectively. The individual antisera showed comparable levels of TB activity (Table 3) for the two strains irrespective of polymorphism at amino acid positions 254, 304 and 322 in the Pfs48/45 portion of R0.10C, suggesting that anti-R0.10C IgG act in a strain-independent manner to control parasite fertility in the infected mosquito. Furthermore, the TB activity for the alum-immunized rats is higher (76–100 %; EC50 titers: 1250–3622) compared with the alum/GLA group (0–72 %; EC50 titers: 196–446) tested at a dilution of 1/27 for both strains.

**Table 3:** Comparison of percentage Transmission-Reducing Activity using 2 *Pf* strains of different geographical origin and sera from rats immunized with R0.10C Alum and Alum/GLA formulation

Group	Rat	NF54			NF135		
		Inf	Mean (SD)	TRA, %	Inf	Mean (SD)	TRA, %
Alum	1	16	7.35 (6.23)	82	17	6.60 (5.99)	76
	2	17	4.45 (4.00)	89	6	0.60 (1.14)	98
	3	1	0.05 (0.22)	100	0	0.0 (0.0)	100
	4	16	4.30 (4.71)	89	10	0.90 (1.21)	97
	5	17	3.20 (2.86)	92	4	0.39 (0.78)	99
Alum/GLA	6	18	27.7 (14.7)	31	17	7.70 (6.87)	72
	7	19	38.8 (23.3)	3	20	15.4 (9.2)	45
	8	20	41.7 (25.9)	0	18	27.1 (15.2)	3
	9	20	17.8 (8.1)	55	18	10.0 (5.85)	64
Pre sera		35/35	39.9 (23.6)	0	40/40	27.8 (12.6)	0

Day 42 sera (1:27 diluted) were tested in the SMFA. Seven days after membrane feed, the number of infected mosquitoes and oocyst density were determined. Sera: rat serum from R0.10C-immunized group; Control: feeder with pooled pre-bleed serum (day 0) of the group. Inf: total infected from 20 dissected mosquitoes; SD: Standard deviation; TRA, %: percentage of Transmission Reducing Activity. NF54: African strain and NF153: Cambodian strain

## Discussion

The development of a TBMV is an attractive goal because it holds the potential to accelerate elimination or even eradication of *P. falciparum* malaria. The inclusion of a TBMV in these efforts would require a highly immunogenic vaccine formulation with the capacity to induce high levels of TB antibodies lasting for at least one transmission season. This is particularly important since several malaria antigens have proved to be poorly immunogenic in adjuvants for human use [32, 33]. Recently, a new multi-stage vaccine candidate (R0.10C) was developed consisting of the N-terminal domain of GLURP (R0) fused in frame to a functional domain of *Pf*s48/45 (10C) [19]. The potential importance of increasing the overall immunogenicity of R0.10C and the *Pfs*48/45 domain in particular, is suggested by the direct relationship between anti-R0.10C antibody titre and biological activity in the SMFA [19]. In the present study, a series of adjuvant formulations in small rodents were investigated for their ability to enhance antibody responses against sexual blood stages. Antisera were also investigated for their ability to control parasite fertilization in the SMFA. It is evident that both Alum and SE/GLA formulations elicited strong TB immunity with five out of five animals showing >99 % TRA. In contrast, R0.10C formulated in Alum/GLA or in AbISCO was slightly less efficient with four out of five animals seroconverting to functional antibody titres. All mice and rat sera were tested at a 1/9 dilution. Testing rat sera from the Alum and Alum/GLA group at dilution of 1/27 still resulted in strong TB activity for the Alum group but not for the Alum/GLA group, showing that Alum alone is a better adjuvant for functional antibodies.

The analysis of specific antibody responses against the 10C domains of R0.10C suggests that the Alum formulation lead to the highest levels of 10C-specific antibodies compared to all other formulations (Fig. 2d). In contrast, an oil-in-water emulsion formulation of GLA led to relatively lower levels of 10C-specific IgG in mice, but not in rats (Fig. 2b). The discrepancy between the antibody titer measured in the R0.10C and MBP.10C ELISAs is due to antibodies against the R0 region of R0.10C. Since recombinant MBP.10C only share the *Pf*s48/45-10C region with the R0.10C fusion protein, MBP.10C was used for the detection of 10C-specific antibodies. MBP.10C was included in the present study to allow comparison with previously published data [6]. Re-testing in a larger group of animals and/or using the 10C part alone may help to clarify whether there is indeed a specific difference.

The identification and characterization of TB epitopes of *Pf*s48/45 have been facilitated by the availability of mAbs with TB activity [8, 12, 13]. In the process of developing a *Pf*s48/45-based TBMV, a series of mAbs was used against conformation-dependent epitopes for the characterization of recombinant *Pf*s48/45 [6, 13]. Here, it has demonstrated that a R0.10C antigen preparation which is correctly folded with respect to epitope 1 of *Pfs*48/45 elicits high levels of antibodies against this epitope in rats with the capacity to inhibit parasite growth in the mosquito when adjuvanted in Alum, Alum/GLA, SE/GLA, or AbISCO. Sera which inhibit a high level of TB activity (TRA) also has a low mosquito infection rate (Tables 1, 2, 3). Adjuvanted with Alum resulted in the highest TB activity (Table 3). Recently, Singh and others [20] show that R0.6C (only epitope 1 of *Pfs*48/45) did also give high TB activity. Further studies must be performed combining R0.10C and R0.6C for immunogenicity data.

The adjuvants tested here (Alum and SE with/without addition of GLA or AbISCO) are all applicable for human use [28, 34]. Aluminium salts are the most commonly used adjuvants in clinical trials and they have the reputation of being safe and give high antibody titres and long-lasting antibody responses [34]. Whether R0.10C/Alum is also a strong immunogen for induction of functional antibodies in humans remains to be determined.

In summary, the *Lactococcus lactis* expression platform was used for production of correctly folded *Pf*s48/45. Since Alum has been used in past malaria vaccine trials of GLURP-based vaccine candidates [21, 35], a further vaccination study with the R0.10C antigen in combination with Alum alone can be pursued.

## Conclusions

An investigation of immune responses to R0.10C using different adjuvants resulted in good antibody response with TB activity. A clinical trial with the R0.10C antigen in combination with Alum appears to be a favourable option.
